# Comparison of lung ultrasound and chest radiography for detecting pneumonia in children: a systematic review and meta-analysis

**DOI:** 10.1186/s13052-024-01583-3

**Published:** 2024-01-23

**Authors:** Yalong Yang, Yuexuan Wu, Wen Zhao

**Affiliations:** 1 Department of Pediatrics, General Hospital of Ningxia Medical University, Yinchuan, 750002 China; 2 Ningxia Medical University, Yinchuan, 750004 China

**Keywords:** Lung ultrasound, Chest X-ray, Pneumonia, Children, Meta-analysis

## Abstract

**Background:**

Lung ultrasound (LUS) is recommended as a reliable diagnostic alternative to chest X-ray (CXR) for detecting pneumonia in children.

**Methods:**

PubMed, Embase, and Cochrane Library databases were used to identify eligible studies from their inception until April 2023. The investigated diagnostic parameters included sensitivity, specificity, positive likelihood ratio (PLR), negative likelihood ratio (NLR), diagnostic odds ratio (DOR), and area under the receiver operating characteristic curves (AUC).

**Results:**

Twenty-six studies involving 3,401 children were selected for meta-analysis. The sensitivity, specificity, PLR, NLR, DOR, and AUC of LUS for detecting pneumonia in children were 0.95, 0.92, 12.31, 0.05, 108.53, and 0.98, respectively, while the sensitivity, specificity, PLR, NLR, DOR, and AUC of CXR were 0.92, 0.93, 24.63, 0.08, 488.54, and 0.99, respectively. The sensitivity of LUS was higher than that of CXR for detecting pneumonia in children (ratio: 1.03; 95% CI: 1.01–1.06; *P* = 0.018), whereas the DOR of LUS was significantly lower than that of CXR (ratio: 0.22; 95% CI: 0.06–0.85; *P* = 0.028).

**Conclusions:**

This study found that the diagnostic performance of LUS was comparable to that of CXR for detecting pneumonia, and the sensitivity of LUS was superior to that of CXR.

**Supplementary Information:**

The online version contains supplementary material available at 10.1186/s13052-024-01583-3.

## Background

Pneumonia is the main cause of hospitalization and the leading cause of death in children aged < 5 years worldwide [1]. Early diagnosis and timely treatment are important for reducing morbidity and mortality [2]. The symptoms of pneumonia are non-specific in children, and there is no single test with a high sensitivity and specificity for diagnosing pneumonia. Clinicians diagnose pneumonia in children in resource-limited settings using the World Health Organization criteria; however, the sensitivity and specificity are low, which results in misdiagnosis and overtreatment [3, 4]. Chest computed tomography is regarded as the gold standard for detecting pneumonia; however, its routine use is restricted by cost, accessibility, and radiation exposure [5].

In clinical practice, chest radiography (CXR) is a widely used imaging modality for diagnosing pneumonia [6]. However, the routine use of CXR is restricted by some diagnostic and technical limitations, including the absence of definitive diagnostic criteria and intra- and inter-observer variations [7–9]. Moreover, exposure to ionizing radiation in children could increase the risk of cancer later in life [6, 10, 11]. Lung ultrasound (LUS) is radiation-free, portable, and inexpensive, which can be conducted at the point of care. Furthermore, the portable ultrasonography machines was easier obtained, which raises the potential of LUS for diagnostic methods in remote settings. It could identify complications of pneumonia and is widely used for the diagnosis and management of pneumonia in children [12, 13]. However, whether the diagnostic performance of LUS and CXR for pneumonia in children is comparable remains unclear. Therefore, the current systematic review and meta-analysis was performed to compare the diagnostic performance of LUS with that of CXR in detecting pneumonia in children.

## Methods

### Data collection

This study was performed according to the Preferred Reporting Items for Systematic Reviews and Meta-Analysis Statement [14]. The study protocol was registered at the INPLASY register (INPLASY202340071). We searched for studies that presented the diagnostic value of LUS with CXR for diagnosing pneumonia in children, and no restrictions were placed on publication language and status. We systematically searched PubMed, EmBase, and the Cochrane Library to screen eligible studies throughout April 2023, and used ((“pneumonia” [MeSH Terms] OR “pneumonia” [All Fields]) AND (“ultrasound” [MeSH Terms] OR (“ultrasound” [All Fields]) as search terms. The search terms were restricted to “Child: birth-18 years.” We also manually reviewed relevant reference lists, citation searches, and systematic reviews to identify any new eligible studies.

The processes of literature search and study selection were independently performed by two reviewers, and any disagreement between reviewers was resolved by discussion with an additional reviewer. Study was included if they met: (1) participants: all of individuals aged < 18.0 years, and suspected for pneumonia; (2) diagnostic tools: the study had to applied both LUS and CXR as diagnostic tools; (3) gold standard: the gold standard for diagnosing pneumonia should clear report; (4) outcomes: studies reported true positive, false positive, false negative, true negative, or data could be transformed into such; and (5) study design: no restrictions placed on study design, including prospective and retrospective design.

### Data collection and quality assessment

The following variables were independently collected by two reviewers: first author’s name, publication year, country, study design, sample size, number of boys/girls, mean age, setting, pneumonia diagnosis, diagnostic tool, true positive, false positive, false negative, and true negative data. Then, the methodological quality was assessed by the quality assessment of diagnostic accuracy studies-2 (QUADAS-2), which was based on patient selection, index tests, reference standard, and flow and timing; the categories low risk, high risk, and unclear were assigned to each study [15]. Inconsistent results regarding data collection and quality assessment between reviewers were resolved by a third reviewer.

### Statistical analysis

The diagnostic parameters of LUS and CXR were analyzed using true positive, false positive, false negative, and true negative data with a bivariate generalized linear mixed model and a the random-effects model. The calculated outcomes included sensitivity, specificity, positive likelihood ratio (PLR), negative likelihood ratio (NLR), diagnostic odds ratio (DOR), and area under the receiver operating characteristic curves (AUC) [16, 17]. The heterogeneity among studies was evaluated using the *I*^*2*^ and Q statistics, and *I*^*2*^ ≥ 50.0% or *P* < 0.10 was defined as significant heterogeneity [18, 19]. Then, the ratio of sensitivity, specificity, PLR, NLR, DOR, and AUC between LUS and CXR were compared using the random-effects model [16, 17, 20]. Subsequently, subgroup analyses were performed based on country, study design, mean age, and gold standards. A funnel plot with Deeks’ asymmetry test was applied to assess potential publication bias [21]. All reported *P* were 2-sided, and the inspection level for pooled conclusions was 0.05. STATA software (version 12.0 StataCorp, Texas, USA) was used to perform all statistical analyses.

## Results

### Literature search

**Fig. 1:**
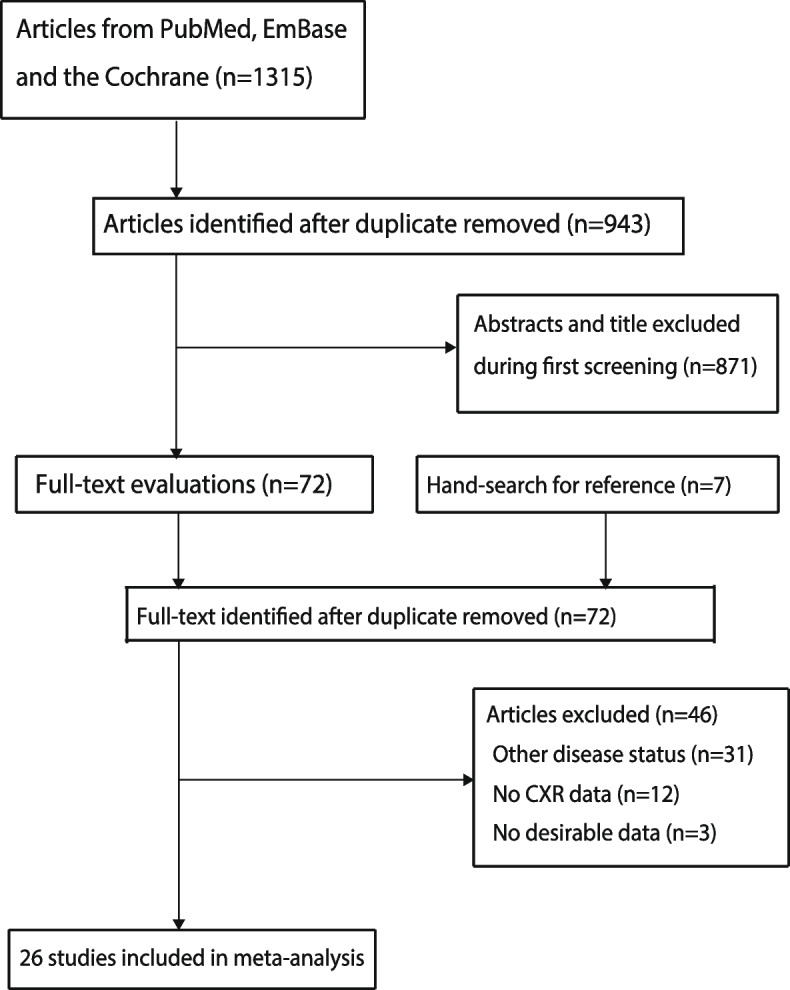
The processes of literature search and study selection

An initial electronic search yielded 1,315 records, and 943 studies were retained after removing duplicate studies. After the title and abstract were reviewed for relevance, 871 studies were removed. The remaining 72 studies were retrieved for detailed evaluations, and 46 studies were excluded because of other diseases (*n* = 31), no CXR data (*n* = 12), and no desirable data (*n* = 3). A total of seven articles were identified by manually reviewing the reference lists of relevant articles, and all of these studies were removed owing to duplicate articles. Subsequently, 26 studies were selected for quantitative meta-analysis [22–47]. The literature search and study selection process are shown in Fig. 1.

### Study characteristics

Table 1 summarizes the baseline characteristics of the included studies and patients. Of the included studies, 22 studies were prospective, and four studies were retrospective. These studies involved 3,401 children, and the sample size ranged from 28 to 641. The mean age of the included children ranged from newborn to 6.5 years. Twenty-one studies were performed in Western countries, and five studies were conducted in Eastern countries. Sixteen studies used clinical criteria to diagnose pneumonia, and the remaining 10 studies used CXR to diagnose pneumonia. The methodological quality of the included studies is shown in Table S1, and the overall quality of the included studies was moderate to high.

**Table 1:** The baseline characteristics of included studies

Study	Region	Study design	Sample size	Boy/girl	Age (years)	Setting	Pneumonia diagnosis	Diagnostic tool	TP	FP	FN	TN
Copetti 2008 [22]	Italy	Prospective	79	37/42	5.1	Emergency department	CXR	LUS	60	0	0	19
								CXR	53	0	7	19
Iuri 2009 [23]	Italy	Prospective	28	17/11	4.5	Pediatric emergency ward	CXR	LUS	22	0	2	4
								CXR	24	0	0	4
Shah 2013 [24]	USA	Prospective	200	112/88	3.0	Emergency departments	CXR	LUS	31	18	5	146
								CXR	36	0	0	164
Caiulo 2013 [25]	Italy	Prospective	102	53/49	5.0	Pediatric departments	Physical and CXR	LUS	88	0	1	13
								CXR	81	0	8	13
Seif 2013 [26]	Egypt	Prospective	75	36/39	Newborn	Neonatal ICU	CXR	LUS	64	4	7	0
								CXR	64	0	11	0
Esposito 2014 [27]	Italy	Prospective	103	56/47	5.6	Pediatric ICU	Physical and CXR	LUS	47	3	1	52
								CXR	48	0	0	55
Liu 2014 [28]	China	Prospective	80	43/37	Newborn	Neonatal ICU	Physical and CXR	LUS	40	0	0	40
								CXR	40	0	0	40
Reali 2014 [29]	Italy	Prospective	107	61/46	4.0	Pediatric departments	Physical and CXR	LUS	76	1	5	25
								CXR	66	2	15	24
Iorio 2015 [30]	Italy	Retrospective	52	NA	3.5	Pediatric ward	BTS guideline	LUS	28	1	1	22
								CXR	25	1	4	22
Urbankowska 2015 [31]	Poland	Prospective	106	NA	4.4	Pediatric ward	Physical and CXR	LUS	71	0	5	30
								CXR	76	0	0	30
Ho 2015 [32]	China	Retrospective	163	91/72	6.1	Pediatric ward	BTS guideline	LUS	147	12	4	0
								CXR	151	0	12	0
Ianniello 2016 [33]	Italy	Retrospective	84	44/40	6.0	Emergency departments	Physical and CXR	LUS	60	0	1	23
								CXR	47	0	14	23
Guerra 2016 [34]	Italy	Prospective	222	108/114	4.9	Pediatric departments	Clinical diagnosed	LUS	207	0	7	8
								CXR	197	0	17	8
Boursiani 2017 [35]	Greece	Prospective	69	27/42	4.5	Emergency departments	Clinical and CXR	LUS	62	0	4	3
								CXR	63	0	3	3
Man 2017 [36]	Romania	Retrospective	81	42/39	6.5	Emergency departments	CXR	LUS	57	15	5	4
								CXR	72	0	0	9
Yadav 2017 [37]	India	Prospective	118	55/63	2.2	Emergency departments	Physical and CXR	LUS	99	6	2	11
								CXR	101	0	0	17
Yilmaz 2017 [38]	Turkey	Prospective	160	NA	3.3	Emergency departments	BTS guideline	LUS	142	4	7	7
								CXR	132	0	17	11
Claes 2017 [39]	Belgium	Prospective	143	77/66	3.4	Emergency departments	CXR	LUS	44	8	1	90
								CXR	45	0	8	90
Samson 2018 [40]	Spain	Prospective	200	116/84	2.5	Emergency departments	Physical and CXR	LUS	74	6	11	109
								CXR	85	0	0	115
Zhan 2018 [41]	Denmark	Prospective	82	47/35	1.5	Pediatric departments	CXR	LUS	33	7	49	75
								CXR	41	0	0	41
Biagi 2018 [42]	Italy	Prospective	87	43/44	5.8	Pediatric departments	Physical and CXR	LUS	25	10	0	52
								CXR	24	8	1	54
Lissaman 2019 [43]	Australia	Prospective	97	47/50	2.4	Emergency departments	CXR	LUS	46	17	4	30
								CXR	44	0	0	17
Bloise 2021 [44]	Italy	Prospective	68	38/30	4.9	Pediatric ward	CXR	LUS	40	1	1	26
								CXR	40	1	1	26
Zhu 2022 [45]	China	Prospective	60	NA	Newborn	Pediatric ward	Physical and CXR	LUS	29	2	1	28
								CXR	28	0	1	1
Don 2022 [46]	Italy	Prospective	641	NA	< 16.0	Pediatric departments	CXR	LUS	575	14	26	26
								CXR	533	5	68	35
Guitart 2022 [47]	Spain	Prospective	194	81/113	0.4	Pediatric ICU	BTS guideline	LUS	76	2	21	95
								CXR	82	46	15	51

### Sensitivity and specificity

**Fig. 2:**
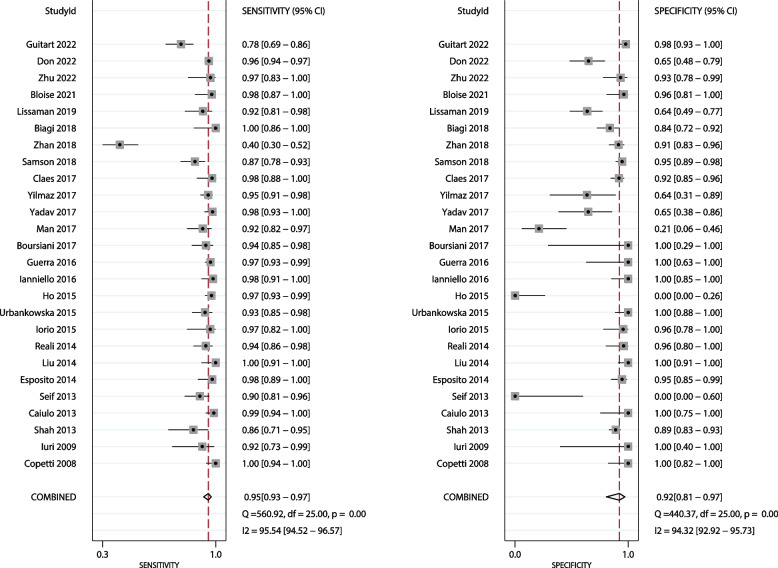
The summary sensitivity and specificity of LUS for detecting pneumonia

The summary sensitivity and specificity of LUS for detecting pneumonia in children were 0.95 (95% CI: 0.93–0.97), and 0.92 (95% CI: 0.81–0.97), while the sensitivity and specificity of CXR were 0.92 (95% CI: 0.90–0.93), and 0.93 (95% CI: 0.91–0.95), respectively (Fig. 2). We noted that the sensitivity of LUS was higher than that of CXR for detecting pneumonia in children (ratio: 1.03; 95% CI: 1.01–1.06; *P* = 0.018), whereas there was no significant difference between LUS and CXR for specificity (ratio: 0.99; 95% CI: 0.90–1.09; *P* = 0.819). Subgroup analyses found that LUS was associated with a higher sensitivity than CXR in most subgroups, whereas no significant difference was observed between LUS and CXR for sensitivity if pooled studies were conducted in Eastern countries, had a mean age < 5.0 years, and used CXR diagnosed pneumonia (Table 2). Moreover, there were no significant differences in specificity between LUS and CXR in all subgroups (Table 2).

**Table 2:** Subgroup analyses for diagnostic performance of US and chest radiography

Parameters	Factors	Subgroups	US	Chest radiography	US vs. chest radiography	* P *value
Sensitivity	Country	Eastern	0.97 (0.95–0.98)	0.94 (0.91–0.97)	1.03 (1.00-1.07)	0.083
		Western	0.95 (0.91–0.97)	0.91 (0.90–0.93)	1.04 (1.01–1.08)	0.019
	Study design	Prospective	0.95 (0.92–0.97)	0.92 (0.91–0.93)	1.03 (1.00-1.06)	0.028
		Retrospective	0.97 (0.93–0.98)	0.89 (0.83–0.93)	1.09 (1.02–1.16)	0.007
	Age (years)	≥ 5.0	0.98 (0.95–0.99)	0.92 (0.88–0.94)	1.07 (1.02–1.11)	0.001
		< 5.0	0.94 (0.90–0.96)	0.92 (0.90–0.93)	1.02 (0.99–1.06)	0.244
	Gold standard	CXR	0.93 (0.85–0.97)	0.91 (0.89–0.93)	1.02 (0.95–1.10)	0.540
		Clinical diagnosed	0.96 (0.94–0.98)	0.92 (0.90–0.93)	1.04 (1.02–1.07)	0.002
	Overall	-	0.95 (0.93–0.97)	0.92 (0.90–0.93)	1.03 (1.01–1.06)	0.018
Specificity	Country	Eastern	0.76 (0.10–0.99)	1.00 (0.95-1.00)	0.76 (0.24–2.40)	0.644
		Western	0.94 (0.84–0.98)	0.93 (0.91–0.94)	1.01 (0.93–1.09)	0.790
	Study design	Prospective	0.93 (0.85–0.97)	0.93 (0.91–0.95)	1.00 (0.93–1.07)	1.000
		Retrospective	0.70 (0.01-1.00)	0.98 (0.90-1.00)	0.71 (0.07–7.15)	0.775
	Age (years)	≥ 5.0	0.94 (0.24-1.00)	0.96 (0.91–0.98)	0.98 (0.48-2.00)	0.954
		< 5.0	0.92 (0.83–0.96)	0.93 (0.91–0.94)	0.99 (0.92–1.07)	0.776
	Gold standard	CXR	0.83 (0.55–0.95)	0.99 (0.97–0.99)	0.84 (0.64–1.10)	0.212
		Clinical diagnosed	0.95 (0.84–0.99)	0.89 (0.86–0.92)	1.07 (0.98–1.17)	0.150
	Overall	-	0.92 (0.81–0.97)	0.93 (0.91–0.95)	0.99 (0.90–1.09)	0.819
PLR	Country	Eastern	4.05 (0.33–50.33)	19.70 (4.85–79.94)	0.21 (0.01–3.65)	0.281
		Western	15.06 (5.82–38.97)	25.30 (8.07–79.29)	0.60 (0.13–2.63)	0.494
	Study design	Prospective	13.40 (6.28–28.60)	24.77 (7.98–76.94)	0.54 (0.14–2.11)	0.377
		Retrospective	3.22 (0.08-130.92)	23.08 (5.92–89.99)	0.14 (0.00-7.19)	0.327
	Age (years)	≥ 5.0	16.22 (0.41-634.34)	23.03 (5.84–90.71)	0.70 (0.01–35.49)	0.861
		< 5.0	11.77 (5.39–25.68)	24.67 (6.15–99.03)	0.48 (0.10–2.35)	0.363
	Gold standard	CXR	5.43 (1.74–16.87)	29.37 (10.82–79.73)	0.18 (0.04–0.84)	0.029
		Clinical diagnosed	20.79 (5.64–76.64)	19.86 (5.08–77.58)	1.05 (0.16–6.91)	0.962
	Overall	-	12.31 (4.70-32.23)	24.63 (8.63–70.26)	0.50 (0.12–2.07)	0.340
NLR	Country	Eastern	0.04 (0.01–0.11)	0.03 (0.00-0.45)	1.33 (0.08–21.44)	0.839
		Western	0.06 (0.03–0.10)	0.09 (0.06–0.13)	0.67 (0.33–1.36)	0.267
	Study design	Prospective	0.05 (0.03–0.09)	0.07 (0.05–0.11)	0.71 (0.36–1.40)	0.329
		Retrospective	0.05 (0.01–0.31)	0.12 (0.04–0.40)	0.42 (0.05–3.29)	0.407
	Age (years)	≥ 5.0	0.02 (0.01–0.05)	0.08 (0.03–0.20)	0.25 (0.07–0.87)	0.029
		< 5.0	0.07 (0.04–0.12)	0.08 (0.05–0.12)	0.88 (0.43–1.77)	0.709
	Gold standard	CXR	0.08 (0.03–0.21)	0.06 (0.02–0.12)	1.33 (0.36-5.00)	0.670
		Clinical diagnosed	0.04 (0.02–0.07)	0.09 (0.05–0.15)	0.44 (0.19–1.02)	0.056
	Overall	-	0.05 (0.03–0.08)	0.08 (0.05–0.12)	0.63 (0.32–1.21)	0.161
DOR	Country	Eastern	81.92 (11.48-584.54)	667.85 (63.42-7032.51)	0.12 (0.01–2.63)	0.180
		Western	117.11 (50.77-270.15)	456.63 (136.86-1523.53)	0.26 (0.06–1.11)	0.069
	Study design	Prospective	125.88 (60.27–262.90)	531.40 (155.82-1812.26)	0.24 (0.06–0.99)	0.049
		Retrospective	41.35 (1.21-1415.91)	234.68 (46.36-1187.99)	0.18 (0.00-8.59)	0.381
	Age (years)	≥ 5.0	169.74 (12.07-2386.56)	359.58 (109.34-1182.51)	0.47 (0.03–8.57)	0.612
		< 5.0	96.89 (46.24-203.02)	497.45 (131.40-1883.28)	0.19 (0.04–0.89)	0.035
	Gold standard	CXR	36.90 (12.49-109.05)	904.64 (179.00-4572.05)	0.04 (0.01–0.29)	0.001
		Clinical diagnosed	212.69 (97.44-464.25)	344.23 (76.84-1542.09)	0.62 (0.11–3.35)	0.577
	Overall	-	108.53 (51.30-229.61)	488.54 (160.82-1484.16)	0.22 (0.06–0.85)	0.028
AUC	Country	Eastern	0.97 (0.95–0.98)	0.99 (0.97-1.00)	0.98 (0.96-1.00)	0.066
		Western	0.98 (0.96–0.99)	0.99 (0.98-1.00)	0.99 (0.97–1.01)	0.280
	Study design	Prospective	0.98 (0.97–0.99)	0.99 (0.98-1.00)	0.99 (0.98-1.00)	0.166
		Retrospective	0.97 (0.95–0.98)	0.99 (0.97-1.00)	0.98 (0.96-1.00)	0.066
	Age (years)	≥ 5.0	0.99 (0.97–0.99)	0.99 (0.98-1.00)	1.00 (0.98–1.01)	0.803
		< 5.0	0.97 (0.96–0.98)	0.99 (0.98-1.00)	0.98 (0.97–0.99)	0.006
	Gold standard	CXR	0.96 (0.93–0.97)	0.99 (0.99-1.00)	0.97 (0.95–0.99)	0.004
		Clinical diagnosed	0.98 (0.97–0.99)	0.98 (0.96-1.00)	1.00 (0.98–1.02)	1.000
	Overall	-	0.98 (0.96–0.99)	0.99 (0.98-1.00)	0.99 (0.97–1.01)	0.280

### PLR and NLR

The summary PLR and NLR of LUS for detecting pneumonia were 12.31 (95% CI: 4.70-32.23), and 0.05 (95% CI: 0.03–0.08), while the PLR and NLR of CXR for diagnosing pneumonia were 24.63 (95% CI: 8.63–70.26), and 0.08 (95% CI: 0.05–0.12), respectively (Figure S1). There were no significant differences between LUS and CXR for PLR (ratio: 0.50; 95% CI: 0.12–2.07; *P* = 0.340) and NLR (ratio: 0.63; 95% CI: 0.32–1.21; *P* = 0.161). Subgroup analyses found that LUS was associated with a lower PLR than CXR if pooled studies used CXR as the gold standard. Moreover, LUS was associated with a lower NLR than CXR if the mean age of the children was ≥ 5.0 years (Table 2).

### DOR

We noted that the summary DOR of LUS for detecting pneumonia was 108.53 (95% CI: 51.30-229.61), while the DOR of CXR for diagnosing pneumonia was 488.54 (95% CI: 160.82-1484.16) (Figure S2). The comparison results indicated that the DOR of LUS for detecting pneumonia was lower than that of CXR (ratio: 0.22; 95% CI: 0.06–0.85; *P* = 0.028). Subgroup analyses indicated that LUS was associated with a lower DOR as compared with CXR when pooled prospective studies, the mean age of children was < 5.0 years, and CXR was used as the gold standard to diagnose pneumonia (Table 2).

### AUC

**Fig. 3:**
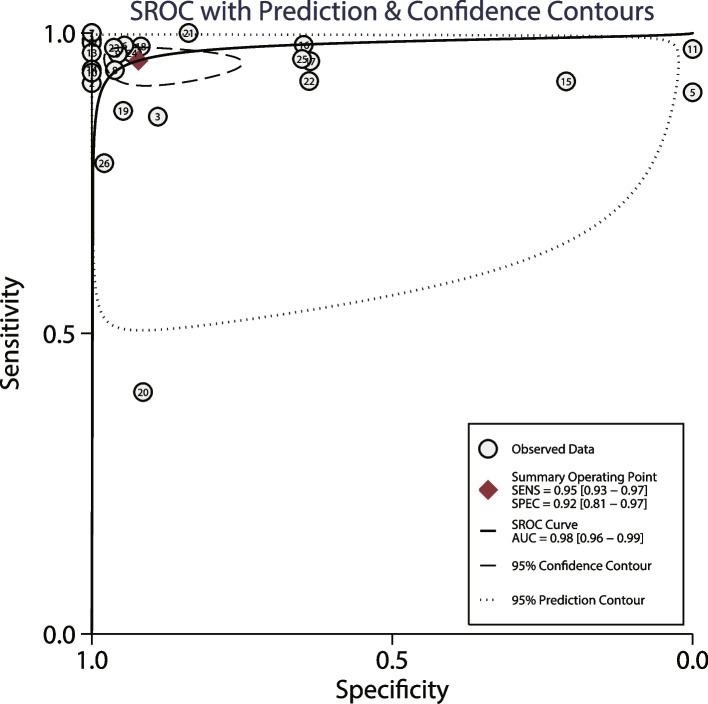
The area under the receiver operating characteristic curves of LUS for detecting pneumonia

The AUC of LUS for detecting pneumonia in children was 0.98 (95% CI: 0.96–0.99), while the AUC of CXR for diagnosing pneumonia in children was 0.99 (95% CI: 0.98-1.00) (Fig. 3). There was no significant difference between LUS and CXR for AUC (ratio: 0.99; 95% CI: 0.97–1.01; *P* = 0.280). Subgroup analyses found that LUS was associated with a lower AUC than CXR when the mean age of children was < 5.0 years, and CXR was applied as the gold standard to diagnose pneumonia (Table 2).

### Publication bias

The publication bias of LUS for detecting pneumonia in children is shown in Figure S3, and the Deeks’ asymmetry test suggested no significant publication bias (*P* = 0.78).

## Discussion

Our study found that the diagnostic values of LUS and CXR were relatively good for detecting pneumonia in children. Moreover, we noted that LUS was associated with a higher sensitivity and lower DOR for detecting pneumonia than CXR. However, we did not find any differences between LUS and CXR for specificity, PLR, NLR, and AUC. Finally, the diagnostic performance between LUS and CXR could be affected by study design, mean age of children, and gold standard for diagnosing pneumonia.

The diagnostic performance of LUS has been investigated in several systematic reviews and meta-analyses [13, 48–51]. Orso et al. identified 17 studies and found that the diagnostic performance of LUS was relatively higher, although these results were restricted by reliable reference standard [48]. Tsou et al. identified 25 studies and found that LUS could accurately detect pneumonia in children, and the performance of LUS could be affected by experienced sonographers [49]. Pereda et al. identified five studies and found that LUS could be considered an imaging alternative for detecting pneumonia in children; however, this conclusion was restricted by unstable results [13]. Xin et al. identified eight studies and supports using LUS for detecting pneumonia in children, and the most common clinical signs of LUS were pulmonary consolidation, positive air bronchogram, abnormal pleural line, and pleural effusion [50]. However, these studies only provided a summary of the diagnostic performance of LUS for detecting pneumonia in children, and the diagnostic value between LUS and CXR was not directly compared [13, 48–50]. Most recently, a meta-analysis conducted by Yan et al. identified 22 studies and suggested that LUS could be regarded as a reliable, valuable, and alternative diagnostic tool to CXR for detecting pneumonia in children [51]. However, this study had several shortcomings, including mistakes on data abstraction, an absence of direct comparison results, and no investigation on the diagnostic performance of LUS versus CXR in study or children with specific characteristics.

Our study found that the diagnostic performance of LUS was relatively high for detecting pneumonia in children, which was consistent with prior meta-analyses [13, 48–51]. We also noted that the diagnostic performance of LUS and CXR for detecting pneumonia in children was comparable. Furthermore, the sensitivity of LUS was higher than that of CXR, which suggests that LUS could differentiate more pneumonia cases, and the prognosis of pneumonia in children could improve. Although CXR is inexpensive and quick, it has a poor ability to distinguish alveolar and interstitial pneumonia. Additional shortcomings of CXR include ionizing radiation and inter-observer agreement [52–54]. The use of LUS can monitor disease progression without exposure to ionizing radiation. Studies have already demonstrated that the use of LUS could shorten emergency department stays, lower financial costs, and reduce complications related to invasive procedures [55–57].

Subgroup analyses found that the diagnostic performance of LUS and CXR for detecting pneumonia in children could be affected by study design, mean age of children, and the gold standard used for diagnosing pneumonia. Several reasons could explain these results: (1) the study design is significantly related to intrinsic biases, and inevitable limitations for retrospective studies include selection and recall biases. Moreover, most included studies were designed as prospective; thus, the pooled conclusions based on retrospective studies were not stable; (2) the diagnostic performance of LUS in children was higher than that in adults for detecting pneumonia [50, 58]. Our study found that LUS was superior to CXR for children aged 5.0 years or older, while the diagnostic performance of LUS was lower than CXR for children aged less than 5.0 years; and (3) numerous included studies applied CXR as the gold standard for detecting pneumonia, and the diagnostic value of CXR may have been overestimated.

This study had some limitations. First, the analysis was based on prospective and retrospective studies, and the pooled conclusions could be affected by uncontrolled selection, recall, and confounding biases. Second, the sonographer’s experience could have affected the diagnostic performance of LUS. Third, the gold standard for diagnosing pneumonia varies across the included studies, which could affect the diagnostic value of LUS and CXR. Fourth, the severity of pneumonia differed across the included studies, which could have affected the complexity of detecting pneumonia in children. Finally, the inherent limitations of meta-analyses based on published data include inevitable publication bias and restricted detailed analyses.

## Conclusions

Both LUS and CXR showed high diagnostic performance in detecting pneumonia in children, and the diagnostic parameters were comparable in terms of specificity, PLR, NLR, and AUC. Moreover, we noted that LUS was associated with higher sensitivity and lower DOR for detecting pneumonia in children than CXR. Exploratory analyses found the diagnostic value of LUS were lower than CXR for detecting pneumonia in children less than 5.0 years. Thus, the LUS should be recommended for detecting pneumonia in older children. Further large-scale prospective studies should be performed to compare the diagnostic value of LUS with CXR for detecting pneumonia in children with specific characteristics.

### Supplementary Information

**Additional file 1: Table S1. **The methodological quality of included studies.

**Additional file 2: Figure S1.** The summary PLR and NLR of LUS for detecting pneumonia.

**Additional file 3: Figure S2. **The summary DOR of LUS for detecting pneumonia.

**Additional file 4: Figure S3. **The publication bias of LUS for detecting pneumonia.

## Data Availability

The datasets used and/or analysed during the current study are available from the corresponding author on reasonable request.

## Abbreviations

LUS: Lung ultrasound
CXR: Chest X-ray
PLR: Positive likelihood ratio
NLR: Negative likelihood ratio
DOR: Diagnostic odds ratio
AUC: A rea under the receiver operating characteristic curves
