# Supplementary material for: Comparison of lung ultrasound and chest radiography for detecting pneumonia in children: a systematic review and meta-analysis
Source: Ital J Pediatr. 2024 Jan 23;50:12. doi: 10.1186/s13052-024-01583-3 (PMC10804756; doi:10.1186/s13052-024-01583-3)
Supplement: Supplementary file 1 — Additional file 1: Table S1. The methodological quality of included studies. [file 13052_2024_1583_MOESM1_ESM.docx]

Table S1. The methodological quality of included studies

| Study | Risk of bias | | | | | Applicability concerns | | | |
| --- | --- | --- | --- | --- | --- | --- | --- | --- | --- |
|  | Patient selection | Index test: US | Index test: CXR | Reference standard | Flow and timing | Patient selection | Index test: US | Index test: CXR | Reference standard |
| Copetti 2008 [22] | Unclear | Low | Low | Unclear | Low | Low | Low | Low | Unclear |
| Iuri 2009 [23] | Low | Low | Low | Low | Low | Low | Low | Low | Low |
| Shah 2013 [24] | Low | Low | Low | Unclear | Unclear | Low | Low | Low | Unclear |
| Caiulo 2013 [25] | Low | Low | Low | Low | Unclear | Low | Low | Low | Low |
| Seif 2013 [26] | High | High | High | Low | High | Low | Low | Low | Low |
| Esposito 2014 [27] | Low | Low | Low | Unclear | Low | Low | Low | Low | Unclear |
| Liu 2014 [28] | Unclear | Low | Low | Unclear | Unclear | Low | Low | Low | Unclear |
| Reali 2014 [29] | Low | Low | Low | Low | Low | Low | Low | Low | Low |
| Iorio 2015 [30] | High | High | High | Unclear | Unclear | Unclear | Unclear | Unclear | Unclear |
| Urbankowska 2015 [31] | Low | Low | Low | Unclear | Low | Low | Low | Low | Unclear |
| Ho 2015 [32] | Unclear | Low | Unclear | Unclear | Unclear | Unclear | Low | Low | Unclear |
| Ianniello 2016 [33] | Unclear | Low | Low | Unclear | Unclear | Unclear | Low | Low | Unclear |
| Guerra 2016 [34] | Low | Low | Low | Unclear | Low | Low | Low | Low | Unclear |
| Boursiani 2017 [35] | Unclear | Low | Low | Unclear | Low | Low | Low | Low | Unclear |
| Man 2017 [36] | High | Unclear | Unclear | Unclear | Unclear | Unclear | Low | Low | Unclear |
| Yadav 2017 [37] | Unclear | Low | Low | Unclear | Unclear | Low | Low | Low | Unclear |
| Yilmaz 2017 [38] | Low | Low | Low | Unclear | Low | Low | Low | Low | Unclear |
| Claes 2017 [39] | Low | Low | Low | Unclear | Unclear | Low | Low | Low | Unclear |
| Samson 2018 [40] | Unclear | Low | Low | Unclear | Low | Low | Low | Low | Unclear |
| Zhan 2018 [41] | Low | Low | Low | Unclear | Low | Low | Low | Low | Unclear |
| Biagi 2018 [42] | Unclear | Low | Low | Unclear | Low | Low | Low | Low | Unclear |
| Lissaman 2019 [43] | Unclear | Low | Low | Unclear | Low | Low | Low | Low | Unclear |
| Bloise 2021 [44] | Unclear | Low | Low | Unclear | Low | Low | Low | Low | Unclear |
| Zhu 2022 [45] | Unclear | Low | Low | Unclear | Low | Low | Low | Low | Unclear |
| Don 2022 [46] | Low | Low | Low | Low | Low | Low | Low | Low | Low |
| Guitart 2022 [47] | Low | Low | Low | Low | Low | Low | Low | Low | Low |
